# Clinical Candidates Targeting the ATR–CHK1–WEE1 Axis in Cancer

**DOI:** 10.3390/cancers13040795

**Published:** 2021-02-14

**Authors:** Lukas Gorecki, Martin Andrs, Jan Korabecny

**Affiliations:** 1 Biomedical Research Center, University Hospital Hradec Kralove, Sokolska 581, 500 05 Hradec Kralove, Czech Republic; lukas.gorecki@fnhk.cz (L.G.); martin.andrs@img.cas.cz (M.A.); 2 Laboratory of Cancer Cell Biology, Institute of Molecular Genetics of the Czech Academy of Sciences, Videnska 1083, 142 00 Prague, Czech Republic

**Keywords:** ATR–CHK1–WEE1 axis, cell-cycle checkpoints, clinical trials, DNA damage response, inhibitors

## Abstract

**Simple Summary:**

Selective killing of cancer cells is privileged mainstream in cancer treatment and targeted therapy represents the new tool with a potential to pursue this aim. It can also aid to overcome resistance of conventional chemo- or radio-therapy. Common mutations of cancer cells (defective G1 control) favor inhibiting intra-S and G2/M-checkpoints, which are regulated by ATR–CHK1–WEE1 pathway. The ATR–CHK1–WEE1 axis has produced several clinical candidates currently undergoing clinical trials in phase II. Clinical results from randomized trials by ATR and WEE1 inhibitors warrant ongoing clinical trials in phase III.

**Abstract:**

Selective killing of cancer cells while sparing healthy ones is the principle of the perfect cancer treatment and the primary aim of many oncologists, molecular biologists, and medicinal chemists. To achieve this goal, it is crucial to understand the molecular mechanisms that distinguish cancer cells from healthy ones. Accordingly, several clinical candidates that use particular mutations in cell-cycle progressions have been developed to kill cancer cells. As the majority of cancer cells have defects in G1 control, targeting the subsequent intra‑S or G2/M checkpoints has also been extensively pursued. This review focuses on clinical candidates that target the kinases involved in intra‑S and G2/M checkpoints, namely, ATR, CHK1, and WEE1 inhibitors. It provides insight into their current status and future perspectives for anticancer treatment. Overall, even though CHK1 inhibitors are still far from clinical establishment, promising accomplishments with ATR and WEE1 inhibitors in phase II trials present a positive outlook for patient survival.

## 1. Introduction

Cancer cells demonstrate characteristics such as sustained proliferative signaling, evading growth suppressors, resisting cell death, induced angiogenesis, activated invasion and metastasis, enabled replicative immortality, deregulated cellular energetics, avoiding immune destruction, genome instability and mutation, and tumor-promoting inflammation, which make it difficult to cure [1]. However, each of these hallmarks provides an opportunity to design specific treatments and agents that can target cancer cells more selectively than conventional chemotherapy. Indeed, a new era of targeted cancer treatment has already begun [2,3]. In this regard, as dysregulation of cell-cycle-controlling proteins causes uncontrolled cell proliferation and enhanced mutagenicity, they represent an interesting area for targeted therapy [4,5]. DNA replication progression is frequently blocked by various DNA lesions (e.g., interstrand crosslinks, T-T dimers, etc.) and ongoing transcription, or is impeded by deoxynucleotide triphosphate (dNTP) depletion and reactive-oxygen-species (ROS) formation [6,7]. Cell-cycle progression needs to be tightly regulated by checkpoints assuring that damaged DNA is repaired correctly, and that DNA replication is completed before entering mitosis. Failure in this can lead to cell death or accelerate mutagenesis to induce tumor formation [8,9,10]. Cell-cycle checkpoints comprise four stages, G1/S (G0), intra‑S, G2/M, and intra‑M, and are regulated by cyclin-dependent kinases (CDKs) complexed with specific subunits called cyclins, whose levels oscillate during the cell cycle [9,10,11]. While G1/S seems to be the most important for assessing cell-fate decisions such as entering division (S‑G2‑M), going to quiescence (G0), or senescence [12,13], insufficient functioning of G1/S is one of the most common features of cancer cells. This aberration is mainly caused by mutations in the tumor-suppressor genes *TP53* or retinoblastoma (*RB*), and/or imbalance in cyclins, CDKs, and their inhibitors [9,14]. Thus, cancer cells are predisposed to rely on their intra‑S and G2/M checkpoints for survival. This is especially true when cancer cells are exposed to chemotherapy or radiotherapeutic DNA damage. A number of kinases are implicated in these controls, protecting cells against replication or mitotic catastrophe, or apoptosis in general [15,16]. Moreover, kinases are also a source of resistance to conventional treatment [17,18]. This review is devoted to inhibitors of kinases, involved in intra‑S and G2/M checkpoints, namely, ATR, CHK1, and WEE1 inhibitors, that entered clinical trials. We reviewed recently presented results from clinical trials to provide insight into their perspectives for anticancer treatment.

## 2. DNA Damage Response and ATR–CHK1–WEE1 Signaling

The DNA damage response (DDR) is the overall cellular response to DNA damage and is manifested by kinase responses that eventually arrest cell-cycle progression and promote DNA repair. Ataxia-telangiectasia and Rad3-related protein (ATR) is one of the main regulators of the DDR, which induce a multitude of mediators and effectors upon various types of DNA damage or replication fork stalling, to resolve the situation. This mainly results in stabilizing the replication fork and preventing its collapse [19,20]. Unresolved stalled forks eventually proceed to irreversible fork breakage and double-strand breaks (DSBs) [21,22]. DSBs are highly severe and potentially lethal events that can be repaired by homologous recombination (HR) operated by ataxia telangiectasia mutated (ATM), or by error-prone nonhomologous end joining (NHEJ) controlled by DNA‑dependent protein-kinase catalytic subunit (DNA‑PKcs) [23,24]. These three kinases (ATR, ATM, and DNA‑PKcs) are the major players of DDR, and belong to the phosphatidylinositol‑3‑kinase-related kinase family (PIKK), representing attractive targets for developing anticancer therapies [17,23,25]. In contrast to the ATM and DNA‑PKcs that are active across the cell cycle, ATR activation is crucial in the late S and G2 phases to ensure proper and replication of the entire genome. Moreover, several studies have revealed that homozygous ATR deletion results in early-embryonic lethal effects, whereas reduction of ATR levels induces resistance to tumor development, as observed in ATR‑Seckel mice [26,27]. Indeed, ATR activity is indispensable for tumor cell lines as they are under permanent replication stress and “backup” pathways for DNA repair are frequently lost, mostly due to *ATM* or *TP53* mutations [28,29].

**Figure 1:**
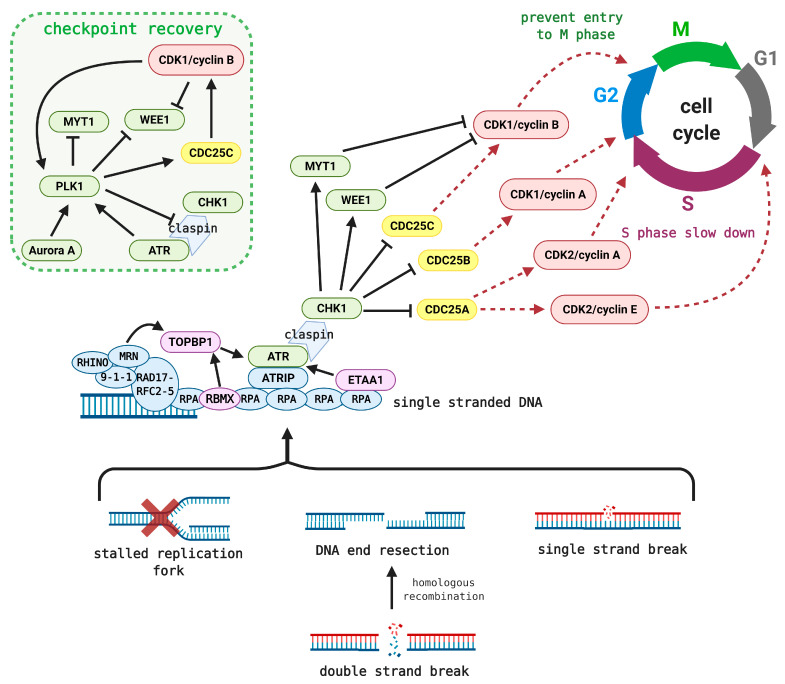
Simplified ATR–CHK1–WEE1 signaling. Stalled replication forks or single and/or double-strand break produce ssDNA that is promptly coated with RPA. ATRIP and ATR are subsequently attached to RPA, after being activated directly by Ewing’s tumor-associated antigen 1 (ETAA1) or in a complex by topoisomerase II-binding protein 1 (TOPBP1) activation. TOPBP1 first needs to be "turned on" by RNA-binding motif protein X-linked (RBMX) or through loaded sensors and mediators such as 9-1-1, RAD17, RFC2-5, MRN, and RHINO [35,36,37]. Activated ATR then phosphorylates and initiates CHK1 via the claspin adaptor. CHK1 marks CDC25 phosphatases for degradation, which further hampers the activation of CDK/cyclin complexes. This results in S-phase slowdown or prevents entry into M phase. Additionally, CHK1 activates the mitotic inhibitors WEE1 and MYT1, which maintain CDK1 in an inactive state. Upon DNA-repair completion, polo‑like kinase 1 (PLK1) phosphorylates claspin, WEE1, and MYT1 to prevent further CDK1 inhibition [38,39,40,41]. Simultaneously, CDC25C phosphatase is activated to cleave the inhibiting phosphorylation on CDK1 [42]. PLK1 is then switched on by aurora A kinase or by ATR-mediated activation through MCM2 [43,44].

As mentioned, ATR activation (Figure 1) starts with DNA damage or, in most cases, from stalled replication fork characterized by extensive single-strand DNA (ssDNA) formation due to polymerase–helicase uncoupling or nucleolytic processing [30]. In normal cells, DNA replication is tightly regulated to not encounter any obstacles. In contrast, DNA replication of precancerous or cancerous cells is often impeded by a shortage of histones or deoxyribonucleotide triphosphates (dNTPs), elevated ROS levels, or increased transcription rates and other topological barriers with both endogenous and exogenous causes [6,31,32]. The danger of replication stress (RS) lies in the formation of fragile ssDNA regions, which are prone to break. Persistent ssDNA is coated with replication protein A (RPA) that directly recruits ATR through the ATR-interacting protein (ATRIP) adaptor. ATR is then allosterically activated by several routes (Figure 1) [19,20,33]. Activated ATR serves as a conductor of many downstream kinases associated with RS response (Figure 1). While ATR precisely phosphorylates several effectors and mediators, a variety of targets are, in turn, phosphorylated by its major downstream partner checkpoint kinase 1 (CHK1), which is switched on via the protein adaptor, claspin [19,34].

**Figure 2:**
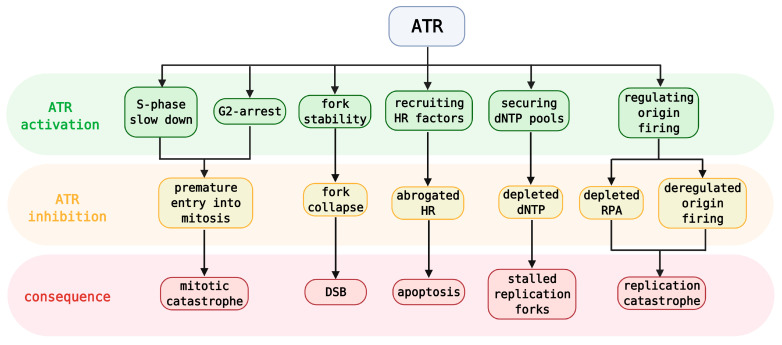
Simplified roles of ATR activation and results of its inhibition with specific consequences. DSB—double-strand break, HR—homologous recombination, RPA—replication protein A.

Apart from the ATR–CHK1 pathway and its role in checkpoint controls, ATR is crucial for protecting replication forks and coordinating DNA replication itself (Figure 2) [20,22,45]. Upon RS, ATR slows replication, induces fork reversal, and limits origin firing, thus preventing collisions with DNA lesions and exhaustion of nucleotides or RPA [46,47]. Deregulated origin firing and extensive RPA exhaustion are prerequisites for replication catastrophe [16,48]. Besides, ATR also secures a sufficient dNTP pool for DNA synthesis avoiding its depletion [49,50]. If the fork collapses and DSBs are formed, ATR helps recruit the factors necessary for HR [51]. Lastly, ATR is associated with nucleotide-excision repair (NER) wherein it phosphorylates the core factor, XPA (Xeroderma pigmentosum complementation group A) [52].

**Figure 3:**
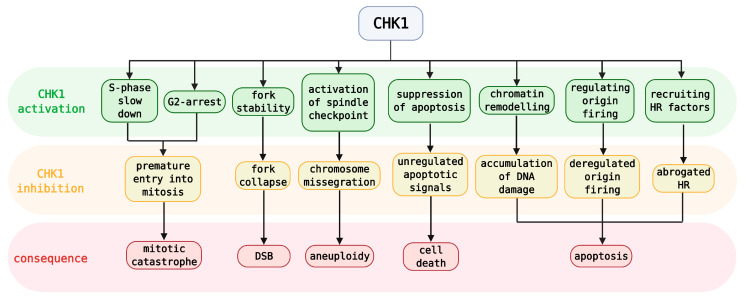
Simplified roles of CHK1 activation and its inhibition with specific consequences. DSB—double-strand break, HR—homologous recombination.

ATR mainly ensures protection and coordination of replication forks, whereas CHK1 is released from the site of damage to further control cell-cycle progression and to summon the subsequent effectors of this pathway (see Figure 1 for CHK1 cell-cycle involvement and Figure 3 for CHK1 activation/inhibition) [53]. The CDC25 phosphatase family is involved in this node. CHK1‑mediated phosphorylation of CDC25 phosphatases leads to their proteasomal degradation; thus, they are no longer able to cleave the inhibitory phosphorylation of CDKs, which results in S-phase slowdown and aberration of G2/M transition [54,55]. In contrast, negative regulation of CDK1 results from inhibitory phosphorylation by WEE1 and MYT1 kinases at Tyr^15^ and Thr^14^, respectively [56,57]. WEE1 is activated by CHK1 and is considered a key mitotic inhibitor that can phosphorylate either CDK1 or CDK2 complexed with cyclin A, B, or E [58,59]. These inhibitory phosphorylations are removed by CDC25 phosphatases [56,60]. The involvement of CDKs and cyclins, as well as CDC25 activity, varies throughout the cell cycle (Figure 1) [61,62].

Additionally, CHK1 maintains S-phase slowdown by abrogating replicon initiation via phosphorylation of treslin or DBF4‑dependent kinase (DDK) [63,64]. Besides phosphorylation of CDC25 phosphatases, the G2 checkpoint is also regulated by transcriptional repression of several genes including *cyclin B1* and *cdk1* [65]. Analogous to ATR, CHK1 function is involved in the stabilization of stalled forks and in their repair by HR upon their collapse. However, though ATR and CHK1 substrates are mostly overlapped, their inhibition may have different impacts on HR [66,67]. CHK1 is involved in chromatin assembly [68] and in mitosis where it affects spindle assembly control and cytokinetic abscission checkpoints by Aurora B phosphorylation [69,70]. Finally, upon excessive accumulation of DNA damage, CHK1 can trigger apoptosis by suppressing caspases 2 and 3 [71].

**Figure 4:**
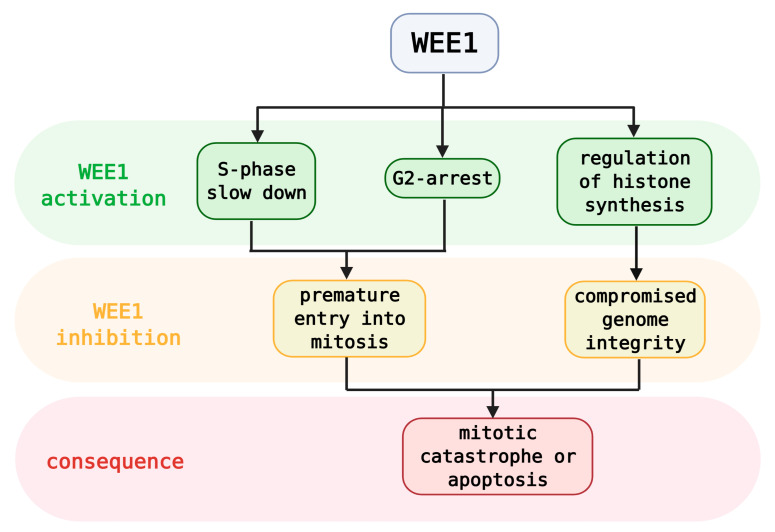
Simplified roles of WEE1 activation and consequences of its inhibition.

In contrast to the broad-acting ATR and CHK1, WEE1 has a rather conservative scale of effectors (Figure 4). However, WEE1 is considered a master regulator of chromatin integrity and a key mitotic gatekeeper. During the S phase, WEE1-mediated phosphorylation of histone H2B at Tyr^37^ terminates histone synthesis, which maintains the correct histone‑DNA stoichiometry. Abrogation of WEE1 function causes mitotic infidelity, chromosome loss, or apoptosis [72,73]. Genomic stability is also maintained by WEE1-mediated protection of stalled-fork integrity, whereas WEE1 inhibition enhances additional replicative stress and DNA damage that further dysregulates cell-cycle progression [74,75]. Finally, upon WEE1 inhibition, cells in the S- or G2‑phase‑arrested state are forced to enter unscheduled mitosis with unrepaired DNA damage, which ultimately results in cell death via mitotic catastrophe [76,77].

**Figure 5:**
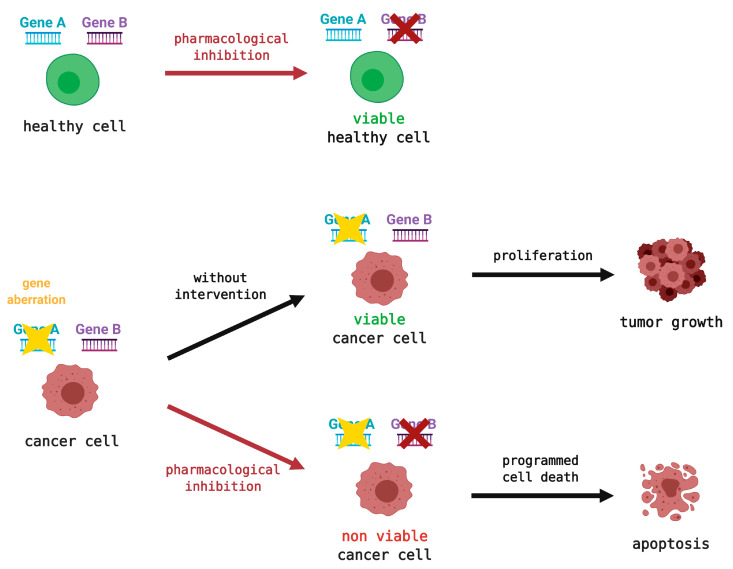
General schematic representation of the synthetic lethality principle. Pharmacological inhibition of one gene will not affect healthy cells reliant on two genes but will demonstrate a lethal effect on cancer cells with the second gene already aberrant. Such cells will undergo programmed cell death, apoptosis. In contrast, a mutation in an untreated cancer cell can enable proliferation and tumor growth. The red cross indicates the possible sites of pharmacological intervention, whereas the yellow star denotes gene mutation.

ATR/CHK1/WEE1 kinases are often elevated in various tumors as they compensate for the lack of other factors, mostly from defective G1/S checkpoints [78,79,80]. Promoted RS rates in cancer cells make the ATR/CHK1/WEE1 kinases a suitable target for anticancer therapy [4,17]. Their inhibition using small-molecule drugs in combination with standard DNA-damaging agents is considered an optimal approach for radio- or chemo-sensitization. These drugs can also exploit events known as "synthetic lethality." Synthetic lethality is described as an aberration of two genetic events resulting in a lethal effect, whereas deviation of either one alone has no significant impact on the cell (Figure 5) [81,82]. For these reasons, the selectivity of these inhibitors is highly appreciated, as they would only target the defective cancerous cells and spare the healthy ones. Olaparib, an FDA-approved poly(ADP-ribose) polymerase inhibitor (PARPi) for treating breast or ovarian cancer with breast cancer susceptibility gene 2 (*BRCA2*)‑deficiency, serves as a proof of this concept [83,84]. Another spark of hope was ignited by the provisional introduction of a CDK4/6‑selective inhibitor (palbociclib), to the market as the first cell-cycle kinase inhibitor [85].

In the following sections, we highlight the clinical potential of targeting the kinases involved in the DDR and in the intra‑S and G2/M checkpoints, namely, ATR, CHK1, and WEE1. We present instances of success with the inhibitors of the titled kinases along with some examples of failure and provide perspectives for their clinical relevance (see Table 1 for the simplified list of drug candidates; full version of Table 1 with detailed information of current clinical trials can be found in Supplementary Material, Table S1). Although CDK inhibitors are downstream targets of the selected pathway, they were omitted from this review, as no selective CDK1 or CDK2 inhibitors have entered clinical trials at this time. For more details about the development and progress of CDK inhibitors, readers are kindly referred to other reviews [2,11,86,87]. Likewise, nonselective and highly promiscuous inhibitors were excluded from this review as their efficacy could not be referred to target a specific pathway.

## 3. Rationale in the Design of Clinical Trials

As preclinical data suggest, rational integration of ATR, CHK1, and WEE1 inhibitors into cancer therapy should be narrowly connected to tumors with elevated RS, or in co-administration with agents that can induce RS. As a result, the effect of ATR, CHK1 and WEE1 inhibitors would enhance the sensitivity of cancer cells, thus potentiating therapeutic efficacy. In line with the aforementioned, several tumors have elevated levels of ATR, CHK1, or WEE1, which also serve as ideal biomarker candidates [88,89,90,91]. Elevated levels of ATR/CHK1/WEE1 are often connected to aberrations in the ATM‑p53 pathway as a compensatory mechanism. The ATM-p53 pathway is one of the major synthetically lethal partners [78,92,93,94], although its chemosensitization effect is not always dependent on p53 functionality [95,96,97]. Other inactivating mutations in genes involved in HR or in DDR (e.g., *ARID1A*, *BRCA1/2*, *ERCC4*, *PTEN*, or *XRCC1*) are also tightly connected to anticancer effects [77,80,98,99,100]. Similarly, RS caused by amplification of oncogenes (MYC, RAS, or Cyclin E1) uses similar logic for the utilization of all three kinase inhibitors [78,101,102,103]. Preclinical in vitro as well as in vivo data clearly favor a combinatorial regimen with standard chemotherapy or radiotherapy for potentiating treatment and minimizing resistance [104,105,106]. Radiotherapy mainly causes DSBs whose repair is dependent on ATM-mediated HR; however, this process also relies on an intact ATR–CHK1 pathway [107,108]. Moreover, ATM-deficient cells are known to be resistant to irradiation, which further encourages the use of ATR/CHK1/WEE1 inhibitors [97,109,110]. These encouraging findings have been shortly translated in clinical investigations.

The majority of these trials deals with ATR/CHK1/WEE1 inhibitors in combination with standard chemotherapy or radiotherapy as an outcome of success in preclinical studies. Trials with standard chemotherapy combinations mainly encompass DNA-crosslinking agents (cisplatin, carboplatin), nucleoside analogues (gemcitabine and capecitabine), topoisomerase (Top) I and II inhibitors (irinotecan, topotecan, etoposide, or doxorubicin), taxane analogues (docetaxel and paclitaxel), folate antimetabolites (pemetrexed), and PARPi (olaparib, veliparib, and niraparib). Some other combinatorial partners have also been utilized, but less frequently; these include the recently approved acalabrutinib (Bruton’s tyrosine-kinase inhibitor), monoclonal antibodies (durvalumab, pembrolizumab, or avelumab), or ralimetinib (p38 mitogen-activated protein-kinase inhibitor), and others [111]. Likewise, different cancer conditions have been considered during the clinical trials. The patients selected were mainly in advanced and extensive stages ≥3, with metastatic, recurrent, and standard treatment-resistant cancers. Moreover, the cancer types were mostly selected based on defects in DDR, including HR-related mutations as well as oncogene amplifications (MYC, RAS, or Cyclin E1).

## 4. ATR Inhibitors in Clinical Trials

There are currently four candidates in clinical trials acting as ATR-kinase inhibitors (Table 1). Two of them were developed by Vertex Pharmaceuticals, Inc., but are presently under maintenance of Merck KGaA (berzosertib and M4344); AstraZeneca PLC and Bayer AG operate with ceralasertib and BAY1895344, respectively [112,113,114,115]. Of these, berzosertib and ceralasertib are the most studied candidates, paving the way for others [116,117]. In total, there are over 50 clinical trials in phases I/II, evaluating these four ATRi. With published results, several of them have fostered high expectations supported by the number and diversity of trials. Berzosertib (IV administration) was shown to be well tolerated in monotherapy or in combination with carboplatin, topotecan, and gemcitabine [118,119,120]. Results from 17 patients enrolled in berzosertib monotherapy showed good tolerability and were recommended the phase II dose (RP2D = 240 mg/m^2^ once or twice a week) without any dose‑limiting toxicities (DLTs). The half-life was estimated at 12.8–18.5 h without any evidence of accumulation [118]. Patients (60 mg/m^2^, once a week) with colorectal cancer including *ATM* loss and *ARID1A* mutation (part of chromatin remodeling complex) achieved a complete response (CR) with progression-free survival (PFS) for 29 months [118]. RP2D in combination therapy with carboplatin had to be reduced to 90 mg/m^2^ (24 h after carboplatin administration) due to hematologic toxicity grades 3/4 (G3/4) at higher doses. Fifteen patients maintained stable disease (SD) as the best response, and one patient with advanced ovarian cancer demonstrated a partial response (PR) even though diagnosed with platinum-refractory and PARP-resistant conditions. Pharmacodynamic analysis showed an initial increase of pCHK1 when treated with carboplatin, and substantial depletion of pCHK1 was observed after 2 h of berzosertib administration [118]. The combination of berzosertib with topotecan was well tolerated up to the highest dose of 210 mg/m^2^ (days 2 and 5 of a 21-day cycle), with only one patient showing a DLT condition (G4 thrombocytopenia). Overall, myelosuppression (G3/4) comparable to topotecan monotherapy was observed without additive toxicities by berzosertib. Reduction of γH2AX (a marker of DNA damage) and probably altered DDR was evident within minutes of berzosertib administration, which subsequently resulted in accrued DNA damage and increased γH2AX levels. Specifically, concomitant administration with topotecan distinctively elevated DNA damage on the following day. Overall, 7/8 patients with SD reached improvement or maintained SD, and 3/5 patients with platinum‑refractory small-cell lung cancer (SCLC) achieved PR or prolonged SD. The best responses were correlated with defective DDR pathways encompassing *ARID1A*, *CHK2,* or *BRCA1* mutations [119].

The results from the randomized phase II trial showed encouraging results for the potential of berzosertib. Patients with recurrent, advanced, and platinum-resistant ovarian cancer were treated with gemcitabine alone or with the combination of gemcitabine and berzosertib (210 mg/m^2^) (36 and 34 patients, respectively). Serious adverse effects (SAEs) G3/4 connected to myelosuppression were observed in ten and nine patients who received gemcitabine and the combination therapy, respectively. In both groups, 13 patients achieved dose reduction, and one patient died due to treatment-related issues. A higher objective response rate was achieved in the gemcitabine alone group; however, the clinical benefit rate as well as the 6 month PFS was better in the combination group. Moreover, higher PFS was especially evident in patients with shorter platinum‑free periods [120]. Regarding ceralasertib, only some preliminary outputs can be drawn from clinical trials. Ceralasertib (P.O.) was well tolerated in monotherapy (RP2D assessed as 160 mg) [121], or in combination with carboplatin (RP2D assessed as 40 mg of ceralasertib), olaparib (RP2D = 160 mg of ceralasertib), and durvalumab (RP2D = 80–240 mg of ceralasertib) [122,123]. Among DLTs (G3/4) thrombocytopenia and neutropenia mostly occurred in combination with carboplatin and olaparib, respectively. Higher responses were evident in ATM-deficient cancers for the carboplatin group, and in *BRCA1/2*-mutated cancers for the olaparib-treated group where responses were independent of ATM status [122,123]. Moreover, PRs and several prolonged SDs were confirmed for monotherapy [121]. Finally, 18 patients with advanced metastatic solid tumors were enrolled to receive BAY1895344 monotherapy. DLTs occurred only at the maximum tested doses and were connected to myelosuppression (G4), and nausea, and fatigue (both G2), whereas the biologically active doses were well tolerated. Objective responses were especially evident in patients with DDR defects including ATM loss/mutation and *BRCA1* mutation [124]. Currently, there is no additional information related to M4344 in clinical trials (there are two phase I trials with recruiting status).

## 5. CHK1 Inhibitors in Clinical Trials

In contrast to ATR, CHK1 inhibitors (CHK1i) are more advanced in clinical trials and have a longer history. This is mainly because ATR is a large protein that is difficult to purify or extract with a challenging kinase assay. Furthermore, the ATR crystal structure has been released only recently [125]. Notably, ATRi development was also delayed by the belief that ATR inhibition would lead to a massive lethal effect in normal proliferative cells [126,127]. These reasons fostered CHK1i development several years earlier.

Seven CHK1i entered clinical trials phases I/II, namely, prexasertib and rabusertib (Eli Lilly&Co); MK-8776 (Merck KGaA); SRA737 (Sierra Oncology); GDC‑0575 (Genentech, Inc.); PF-00477736 (Pfizer Inc.); and AZD7762 (AstraZeneca PLC). Of these, the trials of prexasertib and partly of SRA737 remain active, whereas the other CHKi clinical trials were completed without further succession or were terminated for business and toxicity reasons. For instance, a phase I study of PF-00477736 in combination with gemcitabine was initiated but was eventually terminated for business reasons, as its preliminary results showed objective progression in most of the enrolled patients [128]. The initial phase I study of AZD7762 was completed, but two others were later terminated based on assessment of the risk–benefit profile connected to cardiac toxicity [129,130]. Of 38 patients, only two showed PR in non‑SCLC (NSCLC), and four showed SD for over 12 weeks after administering AZD7762 with gemcitabine [129]. Similarly, another trial was without objective responses with only five patients in SD status as the best response [130]. GDC‑0575 was shown to be relatively safe when administered (P.O.) as a monotherapy in patients with refractory solid tumors. Its combination with gemcitabine only had modest tolerability. DLTs G3/4 occurred frequently resulting in several discontinuations, mostly related to myelosuppression. GDC-0575 showed rapid onset with C_max_ within 2 h and a half‑life around 22.8 h. Gemcitabine administration resulted in increased pCDK1/2, whereas combination with GDC‑0575 (24 h after gemcitabine) decreased these levels, suggesting CHK1 inhibition. However, CHK1 inhibition by GDC‑0575 might not be sufficient due to low doses derived from the drug toxicity. In the combination group, four patients confirmed PR, of whom three had *TP53* mutation. Five patients had SD for more than five months [131]. Merck’s candidate MK‑8776 successfully overcame phase I, showing positive responses in combination with gemcitabine or cytarabine [132,133]. MK‑8776 was well tolerated but demonstrated QT interval prolongation toxicity in both trials. Of 30 patients, two achieved PRs, and 13 exhibited SD (in six cases for over four months) after combination with gemcitabine in advanced solid tumors [132]. In contrast, the combination with cytarabine in relapsed and refractory acute myeloid leukemia (AML) resulted in higher responses wherein 8/24 patients achieved CRs [133]. However, in the follow-up phase II trial, cytarabine monotherapy was slightly more effective than the combination with MK‑8776. The combination group had 5/14 CRs and one PR, whereas the cytarabine group experienced 8/18 CRs and one PR. These results were observed despite the robust escalation of DNA damage (established by assessing γH2AX phosphorylation levels) after the combinatorial approach. MK‑8776 was able to potentiate cytarabine-induced DNA damage at one hour after treatment. Both groups showed similar toxicities for neutropenia and infectious complications. Among the most profound AEs G3/4, the combination therapy manifested QT interval prolongation, hypertension, and gastrointestinal toxicity, whereas cytarabine monotherapy resulted in cardiac, respiratory, renal, and liver toxicities [134]. Although little is known about SRA737 [135,136], it was shown to be well tolerated (P.O., RP2D assessed as 800 mg/day) in monotherapy with DLTs only at the maximum planned dose that included gastrointestinal intolerability and thrombocytopenia. Ninety-three patients with advanced solid tumors expressing favored gene mutations were enrolled for continuing phase II [135]. SRA737 in combination with low doses of gemcitabine revealed no DLTs. In this ongoing phase II trial, 82 patients with advanced solid genetically defined tumors were enrolled, and their preliminary results showed only mild-to-moderate adverse effects. Moreover, SRA737 could potentiate subtherapeutic doses of gemcitabine, and the combination showed moderate clinical benefits [136]. However, more detailed results are eagerly awaited.

The most advanced CHK1i are Eli Lilly’s candidates. In 2013, rabusertib was eventually discontinued after seven completed trials, of which three were mixed phase I/II. The results from phase I showed that rabusertib (IV) exhibited patient-dependent pharmacokinetic variability and only minor accumulation in the body. Rabusertib was well tolerated in combination with pemetrexed (RP2D assessed as 150 mg of rabusertib) or with gemcitabine (RP2D assessed as 170–230 mg of rabusertib) [137,138,139]. LTDs (G3/4) were connected to myelosuppression, fatigue, and diarrhea. Combination with the folic acid antimetabolite pemetrexed was less effective than with gemcitabine in patients with advanced solid malignancies. Of 23 patients in the pemetrexed plus rabusertib group, only one achieved PR (in pancreatic adenocarcinoma), nine showed SD, and 13 displayed progressive disease (PD) [137]. The ongoing phase II study of rabusertib plus pemetrexed in patients with advanced or metastatic NSCLC did not show a complete response. Of 55 patients, only five achieved PR, and 20 had SD. Surprisingly, there was no correlation between *TP53* status and the response [140]. Similarly, patients with advanced NSCLC were treated with a trio‑combination (39 patients) consisting of rabusertib, pemetrexed and cisplatin, or with a dual combination (22 patients) of pemetrexed-cisplatin in a phase II study. Efficacy measured as PFS was significantly evident for the trio combination compared to the dual combination (the median PFS was 4.7 months to 1.5 months). However, seven patients experienced serious thromboembolic events that occurred only in the trio combination group, which hampered further clinical development [141]. Furthermore, moderate results were obtained in the gemcitabine-plus-rabusertib combination (phase I), resulting in one PR, 22 SDs, and 20 PD statuses in a group of 55 patients [138]. A clinical trial conducted in Japan with the same combination revealed four PR, three SD, and six PD out of 15 patients [139]. The follow‑up phase II study did not show any clinical advantage of rabusertib addition to gemcitabine when compared to gemcitabine monotherapy. The overall survival (OS) and PFS was better for gemcitabine than for the combination (the median OS was 8.3 to 7.8, and the median PFS was 5.6 to 3.5, for the monotherapy to the dual combination, respectively), even though the dual combination reached a higher PR (8.8% and 21.5% for mono and the duo, respectively). The severity of adverse effects was comparable for both groups [142]. Overall, only a negligible association was observed between the clinical responses and rabusertib pharmacological actions [138,140,142].

The failures of rabusertib spurred the shift of the focus to prexasertib as the current leading clinical candidate CHK1 inhibitor. Indeed, prexasertib did well in several trials as a monotherapy, mainly in patients with advanced solid tumors. The monotherapy was tolerated with several DLTs where the most common treatment‑related SAE G4 was neutropenia [143,144,145,146]. However, the neutropenia was transient and could be recovered (median duration of six days) without any supporting care [146]. Prexasertib showed minor intra- and inter‑cycle accumulation with a half‑life ranging from 11.4 to 27.1 h [144]. Initially, of 43 patients with squamous cell carcinoma (SCC), two had PR and 15 exhibited SD [144]. In an ongoing phase I study, 101 patients with SCC were enrolled and were given RP2D previously assessed as 105 mg/m^2^ every 2 weeks. The overall clinical benefit rate after 3 months of therapy was SD in 29%, where one patient achieved CR and six had confirmed PR [143]. In the ongoing phase II, 28 women were enrolled with *BRCA*‑wild type (BRCA‑*wt*) recurrent advanced ovarian cancer. Of these, 22 had platinum‑resistant or platinum‑refractory status. Eight of 24 evaluable patients achieved PR, and five had SD; the median PFS was 7.4 months. In a group of platinum‑resistant or platinum‑refractory disease, six of 19 patients showed PR. Moreover, exploratory analysis connected the amplification of cyclin E1 with clinical benefit, whereas no correlation was observed with deficient HR [146]. Similarly, prexasertib showed modest clinical efficacy in the subsequent phase II trial evaluating nine patients with BRCA‑*wt* recurrent triple‑negative breast cancer (TNBC). One patient exhibited PR, and four achieved SD; the median PFS was 86 days [145]. However, the results from a recent phase I study do not favor the combination of prexasertib with ralimetinib (p38 mitogen‑activated protein-kinase inhibitor) in advanced or metastatic tumors, as of nine enrolled patients, three experienced serious DLTs G4, and only one patient had the best overall response of SD [147].

## 6. WEE1 Inhibitor in Clinical Trials

Clinical trials of WEE1 inhibitors (WEE1i) can be designated as "a one-man show" for adavosertib by AstraZeneca PLC (developed by Merck KGaA). The first report regarding adavosertib dates back to 2009 [110,148]. As of now, over 55 trials in phases I/II have been undertaken since 2008, of which 29 are still active. Similar to ATRi and CHK1i, adavosertib has been challenged against a multitude of cancer types such as monotherapy or in combination with various DNA-damaging chemotherapies or radiotherapy. Initial clinical studies investigated whether adavosertib could reach sufficient concentrations in the brain for the treatment of glioblastoma. Indeed, human-derived in vitro systems revealed that adavosertib permeates the blood–brain barrier (BBB) and accumulates in human glioblastoma tissues [149]. In a phase 0 trial enrolling 20 patients with diagnosed glioblastoma, who received a single dose (P.O.) of adavosertib, it was confirmed that adavosertib demonstrates good CNS availability and efficient drug concentration in tumor tissue. Pharmacodynamics indicated a massive rise in DNA damage measured as γH2AX [150]. In general, an ideal adavosertib dosing was considered twice a day for 2.5 days for 2 consecutive weeks in a 21‑day cycle. Its peak plasma concentration ranged from 1 to 8 h, and its half‑life was around 9–12 h [151,152,153,154]. A phase I study of adavosertib monotherapy in 25 patients with advanced solid tumors suggested 225 mg of RP2D (P.O.). Only two patients developed DLTs G4 including myelosuppression and supraventricular tachyarrhythmia; however, these were presumably linked to former therapeutic interventions. Generally, the toxicological profile for RP2D was considered "acceptable." Further analysis of five patients exhibiting *BRCA1/2* mutation showed two confirmed PRs. Moreover, no clinical response on *TP53* mutation was observed. Pharmacodynamic analysis demonstrated a dramatic reduction in p^Tyr15^CDK1 levels and increased γH2AX levels due to augmented DNA damage [151]. Another phase I trial of adavosertib with a fixed-dose combination of docetaxel plus cisplatin in patients with head and neck SCC (HNSCC) showed good tolerability with only DLT diarrhea (G3). Under these conditions, RP2D dosage was determined as 150 mg twice a day for 2.5 days. No G4 SAEs were observed. Of 10 patients, five reached PRs and four reached SDs, favoring this combination before surgical intervention. All enrolled patients had aberrant p53 signaling; thus, no correlations concerning synthetic lethality could be concluded. Pharmacodynamic analysis showed a particular reduction in p^Tyr15^CDK1 after adavosertib administration, which was well correlated with the extent of the responses [152]. The combination of adavosertib with gemcitabine and radiation was tolerated but with several DLTs, including anorexia, nausea, and fatigue. Half of the enrolled patients experienced AEs G3/4. RP2D dosage was assessed as 150 mg/day for P.O. administration. The clinical benefits still exceeded those of the dual combination comprising gemcitabine and radiation. The triple combination including adavosertib, gemcitabine, and radiation provided a median PFS of 9.4 months in 34 enrolled patients. Gemcitabine-augmented levels of p^Tyr15^CDK1 were gratifyingly decreased by adavosertib addition, indicating a sufficient biological response and target inhibition [155]. Finally, a large phase I cohort study of 202 enrolled patients with advanced solid tumors showed the benefits of adavosertib as a monotherapy or as a combination therapy with one of the selected chemotherapeutics: cisplatin, carboplatin, or gemcitabine. Monotherapy was without DLTs, whereas SAEs G3/4 in the combination therapy included diarrhea, fatigue, vomiting, nausea, and myelosuppression. RP2D dosage was assessed as 200 and 225 mg for cisplatin and carboplatin (twice a day for 2.5 days) combinations, respectively, and as 175 mg once a day for 2 days for the combination with gemcitabine. Adavosertib was given in standard regimens at 24 h after chemotherapy, though the authors also suggested a possible multiple-dose regimen when considering the adavosertib pharmacokinetic profile. Overall, of 176 patients, 17 achieved PR, and 94 had SD. Moreover, this study slightly elicited the influence of the *TP53* status, when the response rate was higher in *TP53*‑mutated compared to *TP53*‑wt patients, from 21% to 12%, respectively. A reduction of p^Tyr15^CDK1 levels in postadavosertib biopsies was evident in the combinations with cisplatin and carboplatin. Indeed, gemcitabine co-administration improved the dose regimen [153]. These findings were successively applied to an ongoing phase II study in patients with *TP53*‑mutated refractory or resistant ovarian cancer. In this study, a combination of adavosertib with carboplatin exhibited extensive but manageable toxicity where the most severe DLTs G4 was myelosuppression. Of 21 patients, one achieved CR, eight exhibited PR, and seven had SD as the best response; the median PFS was 5.3 months. Furthermore, two patients that displayed an ongoing response for 31 and 42 months (at data cutoff), both revealed other mutations in Cyclin E, and in BRCA1, MYC, and Cyclin E, respectively. However, no clear correlation of the clinical benefit and p^Tyr15^CDK1 reduction was observed [154].

## 7. Lessons Learned from the Clinical Trials

### 7.1. The Overall Efficiency of ATR/CHK1/WEE1 Inhibitors

Several concluding remarks have already been made from the completed clinical trials; however, the real proof‑of‑concept, outputs from the phase III study, are eagerly awaited [120,154]. Currently, ATRi represent highly attractive drug candidates, which is well documented by the rapid ascendance of new clinical trials. Indeed, three ATRis (berzosertib, ceralasertib, and BAY1895344) have revealed particular clinical benefits in patients. In particular, a completed phase II randomized trial of berzosertib showed valuable clinical outcomes when combined with gemcitabine in patients with recurrent, platinum-resistant advanced ovarian cancer [120]. The results from this trial warranted study continuation; however, the phase III trial has not been initiated yet. In contrast to the glorified ATRi, CHK1i currently stands aside in clinical development. In general, CHK1i exhibited more SAEs than ATR or WEE1 inhibitors, especially, when used in combinatorial regimens. The failure of CHK1i in providing clinical benefit may be attributed to the insufficient inhibition of CHK1 due to the necessary dose reductions resulting from the drug toxicity. Only prexasertib monotherapy was shown to be relatively well tolerated and could be further utilized in clinical development; however, a more in-depth study is required to assess its actual clinical relevance. In contrast, adavosertib was well tolerated in monotherapy and in combinatorial regimens, both yielding specific clinical improvements. However, combinatorial therapies seem to be necessary as adavosertib itself is not that efficient in monotherapy. Moreover, the randomized phase II trial is still missing. We can only speculate why adavosertib that entered clinical trial testing more than 12 years ago did not proceed to phase III. Currently, results from completed clinical trials favor ATR inhibitors over WEE1i and CHK1i. Notably, berzosertib displayed clinical efficacy in platinum-refractory and PARP-resistant conditions, whereas the clinical benefits of prexasertib and adavosertib have been proven in patients with recurrent and platinum‑resistant cancers.

### 7.2. Adverse Effects

The major DLTs and SAEs G4 of ATR/CHK1/WEE1 inhibition were associated with myelosuppression, which is the most frequently occurring adverse effect with anticancer agents. CHK1i was found to be the most toxic of this trinity. Low selectivity of the first-generation CHK1i, imposing CHK2 inhibition as well, is one of the culprits of their escalated toxicity. Indeed, the nonselective inhibition pattern of AZD7762 and MK‑8776 is responsible for their significant cardiotoxicity [129,132,133]. The knowledge gained from the development of these agents resulted in the development of the well-tolerated SRA737 with prevailing CHK1 inhibition and mitigated severe myelosuppression [135,156]. On the contrary, even a selective CHK1 inhibitor combined with DNA-damaging agents could result in SAEs due to a significant effect on healthy cells [55,157]. A more rational application of CHK1i could represent monotherapy or combination with subtherapeutic doses of DNA damaging agents as with prexasertib or SRA737 [136,146].

In contrast, ATR is the apex kinase in this DDR machinery; thus, its inhibition could be "easily" compensated by backup pathways that activate CHK1 in the absence of ATR [158,159]. Moreover, ATR levels in unperturbed cells are relatively low and are elevated only as a response to RS [159,160]. This could suggest good tolerability for healthy tissues but a greater importance of a combination strategy with DNA damaging agents. For instance, monotherapy (berzosertib, ceralasertib, and BAY1895344) resulted in no DLTs with an acceptable level of myelosuppression [118,121,124]. Besides, these three inhibitors are highly selective without potential off-target toxicity. M4344 specifically inhibits CHK1 but little is known about its status in an ongoing trial. ATRi combined with standard chemotherapy afforded dose reductions and excellent tolerability at therapeutic doses [119,120,122,123]. Combinatorial regimens of ATRi with chemotherapy could also benefit from combination with subtherapeutic doses of DNA-damaging agents. Chemotherapeutics such as antimetabolites, crosslinking agents, or topoisomerase inhibitors (e.g., gemcitabine, cisplatin, or topotecan, respectively) can also cause myelosuppression by themselves [161,162,163]; thus, dose reduction might prevent their potential toxic events.

Opposite to ATR/CHK1 inhibition, the role of WEE1 is in the last step before mitosis and its inhibition directly shifts cells to mitosis [103,164]. This presumes effective utilization of WEE1 inhibitors as monotherapeutic agents with a low probability of toxicity issues. Indeed, monotherapy was mostly well tolerated with manageable SAEs such as diarrhea or less frequently, myelosuppression. On the contrary, adavosertib is known to inhibit other kinases, of which PLK1 could have a major influence, and to cause more pronounced AEs [165]. Thus, there is still space for improvement to develop highly selective WEE1 inhibitors with suppressed toxicity.

### 7.3. Targeting Biomarkers

The preliminary preclinical data for ATR, CHK1, and WEE1 inhibitors enabled the prediction of which inhibitor would prosper from a particular genetic aberration (see chapter "Rationale in the design of clinical trials") [104,106,166,167]. Some ATRi successfully translated their presumption from basic research into the clinics, and their clinical responses were mostly dependent on the mutation/loss of ATM [118,122,124]. However, the combination of ceralasertib with olaparib was ATM‑independent and preferably profited from the HR defects represented by *BRCA1/2* mutations [123]. Likewise, berzosertib with topotecan benefited from HR alterations induced by *BRCA1* and *CHK2* mutations [119]. Mutations of tumor suppressor *ARID1A* were clinically confirmed to be synthetically lethal with ATRi [118,119]. However, further comprehensive clinical investigation of these exploratory objectives identifying specific biomarkers for ATRi is still awaited [120].

The *TP53* mutation proved to be a suitable synthetically lethal partner of CHK1i in the clinic [131,145]. In a small cohort study, prexasertib could show clinical efficacy in TNBC even though they were *BRCA‑wt* [145]. In contrast, HR deficiency associated with *BRCA1/2* mutation warranted CHK1i benefit in another trial [143]. RS induction connected to cyclin E1 amplification was demonstrated to enhance the clinical efficacy of CHK1i [143,146]. A study employing rabusertib with pemetrexed indicated no influence of *TP53* status [140]; although, this could be argued about any unsuitable drug combination for exploiting this synthetic lethality as pemetrexed does not directly damage DNA but rather hampers dNTP synthesis [168].

No clear correlation of *TP53* status and clinical benefit was shown after WEE1 inhibition [151,153]. Adavosertib monotherapy revealed a significant improvement in patients with *BRCA1/2* mutation, but clinical response was not dependent on *TP53* status [151]. Only combinatorial regimens displayed a partial response in patients with *TP53* aberration [153]. The best response so far was obtained for the combination of adavosertib with carboplatin amplifying RS inducers, MYC, and cyclin E [154].

### 7.4. Resistance

Olaparib therapy is an example in which various mechanisms of acquired resistance have been reported (e.g., drug efflux, PARP protein mutations, and restoration of HR activity) [169,170]. Related events could also be expected for ATR, CHK1, or WEE1 inhibitors. Rationally, the development of inherent or acquired resistance mainly threatens single-agent therapy. Phases I or II are too "short" to develop resistance in cancer cell lines; however, the underlying mechanism must be considered. For instance, ATR-mediated activation of CHK1 could be compensated by ATM or DNA‑PK backup pathways, and active CHK1 can control events similar to those signaled by ATR [66,158]. Resistance to CHK1 inhibition could also evolve through cellular modifications via downstream cell regulators. Recently, prexasertib resistance has been described in BRCA-*wt* ovarian cancer presumably due to prolonged G2 delay by decreased CDK1/cyclin B activity, saving the cells from mitotic catastrophe [171]. Finally, acquired resistance to WEE1 inhibition by adavosertib has been linked to up-regulation of MYT1 in cancer cells. As WEE1 and MYT1 have functional redundancy in CDK1 inhibition, increased MYT1 expression could serve as a biomarker for WEE1 inhibition resistance [172]. Needless to say, an increased drug efflux could also decrease the antitumor activity of ATR, CHK1, and WEE1 inhibitors.

### 7.5. Optimal Time Scheduling of Combinatorial Regimens

For ATR/CHK1/WEE1 inhibitors, the ideal timeframe of combinatorial regimens was estimated to be 12–24 h after administration of the DNA damaging agent. This schedule clearly corresponds with the peak accumulation of cells in the S phase and follows ATR/CHK/WEE1 activation [110,173,174,175]. However, some deviation in the dose regimen needs to be considered based on the drug pharmacokinetic profiles. As seen in the combination of adavosertib with gemcitabine, the alternative schedule resulted in improved pharmacodynamic outcomes [153,155]. The first dose was taken simultaneously with adavosertib or after 3–4 h, and the second dose was taken after 24 h of gemcitabine infusion [153,155]. Intravenously (I.V.) administered drugs have mostly rapid onset whereas P.O. administered drugs show delayed onset of pharmacological action and may thus demonstrate variable peak plasma concentrations. In general, P.O. administration might be favored as it is the most comfortable for a patient, enabling home treatment without the risk of potential nosocomial infection. As standard chemotherapy is mostly administered through I.V. infusion, P.O. DDR inhibitors could reduce the frequency of necessary hospital visits in combination regimens. In case of a long-term treatment with a single agent, P.O. administration can be considered ideal. DNA damage is mostly measured as Ser^139^ phosphorylation of the histone protein H2AX, which serves as a reliable biomarker of RS and DSBs [176,177,178]. Target inhibition is estimated indirectly based on downstream partners, as levels of pCHK1 for ATRis, and levels of pCDK1 for CHK1 and WEE1 inhibitors [110,131,179]. In summary, clinical trials have confirmed the aforementioned time scheduling and pharmacodynamic analyses have shown elevated levels of DNA damage after treatment with ATR/CHK1/WEE1 inhibitors. However, in some cases, pharmacological target inhibition was not correlated with clinical outcomes. Especially, CHK1 inhibitors did not achieve sufficient inhibition due to toxicity issues, or CHK1 inhibition was not correlated with the overall response [151,153,160,162,164].

## 8. Conclusions

Although the hallmarks of cancer hinder its cure, they can be effectively utilized for selective cancer-cell targeting to ultimately cure the patient. The clinical success of olaparib, the first FDA-approved DDR inhibitor, has strengthened efforts to inspect other DDR kinases and inhibitors. The ATR–CHK1–WEE1 axis is highly implicated in cancer-cell processing as these cells are under constant RS with various defects hampering DNA repair. To underpin the synthetic-lethality approach in combination with DNA-damaging agents, several preclinical data, and their successes have allowed a few drug candidates to enter clinical trial testing. All of these drug candidates are highly potent and selective inhibitors in the low or even sub-nanomolar range; however, only a few of them have exhibited satisfactory clinical outcomes in patients. The results of these trials have indicated the importance of several other factors implicated in cancer treatment. For instance, inhibition selectivity is crucial for eliminating off-target effects and reducing toxicity in general. A large number of kinases with structurally similar ATP‑binding pockets represents a great challenge for developing extremely selective drugs; however, only selective drugs could achieve the desired anticancer effects without exerting any toxicity. The development of novel potential kinase inhibitors thus has to proceed hand‑in‑hand with the improvement of biomarker analysis. Only flawless identification of the patient genome will help fully utilize the targeted cancer treatment. Indeed, some biomarkers for ATR/CHK1/WEE1 inhibitors still have to be verified in order to render the maximum clinical benefits. Proper patient genome and cancer biomarker identification with suitable combinatorial regimens will thus become a new weapon for oncologists to fight with cancer.

## Figures and Tables

**Table 1:** ATR, CHK1, and WEE1 inhibitors that have entered clinical trials. Full version of the table including cancer types and clinical-trial identification (NCT) numbers can be found in Supplementary Material (Table S1).

Target Kinase	Clinical Candidate (Alternative Names)	Ki / IC50 of the Effected Kinases	Administration	Phase	Status
ATR	berzosertib (M6620/VX-970/VE-822)	Ki < 0.2 nM	i.v.	I/II	active
	ceralasertib (AZD6738)	Ki = 4 nM	p.o.	I/II	active
	M4344 (VX-803)	ATR Ki < 0.2 nM CHK1 Ki = 8 nM	p.o.	I	active
	BAY1895344	Ki = 7 nM	p.o.	I	active
CHK1	prexasertib (LY2606368)	CHK1 Ki = 0.9 nM CHK2 Ki = 8 nM	i.v.	I/II	active
	rabusertib (LY2603618)	Ki = 0.9 nM	i.v.	I/II	discontinued
	MK-8776 (SCH099776)	IC_50_ = 3 nM	i.v.	I/II	discontinued
	SRA737	IC_50_ = 1.4 nM	p.o.	I/II	active
	GDC-0575 (ARRY-575)	IC_50_ = 1.2 nM	p.o.	I	discontinued
	PF-00477736	Ki = 0.5 nM	i.v.	I	terminated
	AZD7762	CHK1 IC_50_ = 5 nM CHK2 IC_50_ = 10 nM	i.v.	I	discontinued
WEE1	adavosertib (AZD1775/MK-1775)	WEE1 Ki = 3.2 nM WEE2 Ki = 3.9 nM PLK1 Ki = 3.0 nM	p.o.	I/II	active

i.v.—intravenous; P.O. —per os (oral); K_i_—inhibitory constant; IC_50_—the half-maximal-inhibitory concentration.

## Data Availability

Some datasets generated and analyzed during the current study are available in the ClinicalTrials.gov repository, https://clinicaltrials.gov/ (accessed on 19 January 2021).

## Supplementary Materials

The following are available online at https://www.mdpi.com/2072-6694/13/4/795/s1, Table S1: ATR, CHK1, and WEE1 inhibitors that have entered clinical trials, full version with detailed information.

## Nomenclature

Dictionary Box—List of Clinical Terms, Taken from NIH NCI	
Progression-free survival (PFS)	the length of time during/after the treatment of a disease, that a patient life with the disease without it getting worse
Overall survival (OS)	the length of time from either the date of diagnosis or the start of the treatment of a disease that a patient with the disease is still alive
Response	an improvement related to a treatment
Complete response (CR)	the disappearance of all signs of cancer in response to a treatment
Partial response (PR)	a decrease in the size of a tumor, or in the extent of cancer in the body, in response to a treatment
Stable disease (SD)	cancer that is neither decreasing nor increasing in extent or severity
Progressive disease (PD)	cancer that is growing, spreading, or getting worse
Recommended phase II dose (RP2D)	usually the highest dose with acceptable toxicity, used in subsequent clinical trials.
