# Supplementary material for: Clinical Candidates Targeting the ATR–CHK1–WEE1 Axis in Cancer
Source: Cancers (Basel). 2021 Feb 14;13(4):795. doi: 10.3390/cancers13040795 (PMC7918546; doi:10.3390/cancers13040795)
Supplement: Supplementary file 1 [file cancers-13-00795-s001.pdf]

cancers

MDPI

Supplementary Materials

Clinical Candidates Targeting the ATR-CHK1-WEE1 Axis in Cancer

Lukas Gorecki, Martin Andrs and Jan Korabecny

Table S1. ATR, CHK1, and WEE1 inhibitors that have entered clinical trials, full version with detailed informations.

Clinical Candidate (Alternative Names and Structure)	Study Start	Status	Phase	Cancer Type	Clinical Trial Identification
ATR	December, 2012	completed	I	advanced solid tumor	NCT02157792
	July, 2015	recruiting	I/II	small cell cancers and extrapulmonary small cell cancers	NCT02487095
	September, 2016	recruiting	I	HNSCC	NCT02567422
	August, 2016	active, not recruiting	II	metastatic urothelial cancer	NCT02567409
	July, 2016	recruiting	I	treatment brain metastases from NSCLC, SCLC and neuroendocrine tumors	NCT02589522
	June, 2016	recruiting	I	metastatic solid tumors or tumor that cannot be removed by surgery	NCT02595931
	August, 2016	active, not recruiting	II	recurrent ovarian, primary peritoneal, or fallopian tube cancer	NCT02595892
	November, 2016	recruiting	I	recurrent and metastatic ovarian, primary peritoneal, or fallopian tube cancer	NCT02627443
	January, 2018	active, not recruiting	I	advanced solid tumors	NCT03309150
	October, 2018	active, not recruiting	II	metastatic castration-resistant prostate cancer	NCT03517969
	December, 2018	recruiting	I	oesophageal cancer	NCT03641547
	June, 2020	recruiting	II	progressive, metastatic, or unresectable *TP53* mutant gastric or gastroesophageal junction cancer	NCT03641313
	March, 2019	completed	I	PARPi-resistant ovarian cancer	NCT03704467
	January, 2019	active, not recruiting	II	leiomyosarcoma, osteosarcoma	NCT03718091
	August, 2019	recruiting	II	SCLC and small cell cancers outside of the lungs	NCT03896503
	November, 2019	recruiting	I	chemotherapy-resistant triple-negative and estrogen and/or progesterone receptor positive, HER2 negative breast cancer	NCT04052555
	June, 2020	suspended	I / II	NSCLC	NCT04216316
	March, 2020	recruiting	I / II	DDR deficient metastatic or unresectable solid tumors	NCT04266912
	November, 2013	completed	I	lymphomas	NCT01955668
	July, 2014	active, not recruiting	I	solid tumors refractory to conventional treatment	NCT02223923
	November, 2015	active, not recruiting	II	solid tumors	NCT02576444
	December, 2015	recruiting	I	refractory cancer	NCT02630199
	May, 2015	recruiting	II	NSCLC	NCT02664935
	November, 2016	active, not recruiting	II	platinum refractory extensive stage SCLC	NCT02937818

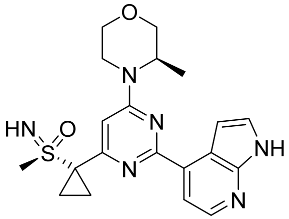 **ceralasertib (AZD6738)**	September, 2017	recruiting	I	HNSCC	NCT03022409
	December, 2016	recruiting	II	advanced breast cancer	NCT03182634
	January, 2018	recruiting	I / II	chronic lymphocytic leukemia	NCT03328273
	February, 2018	active, not recruiting	II	metastatic TNBC	NCT03330847
	December, 2017	recruiting	II	NSCLC	NCT03334617
	October, 2018	active, not recruiting	II	SCLC	NCT03428607
	March, 2018	recruiting	II	recurrent ovarian cancer	NCT03462342
	June, 2018	active, not recruiting	I	relapsed or refractory Non-Hodgkin's lymphoma	NCT03527147
	October, 2019	recruiting	I	advanced or metastatic solid tumors	NCT03669601
	January, 2019	recruiting	II	advanced solid tumors excluding ovarian cancer	NCT03682289
	October, 2019	recruiting	II	chemotherapy resistant TNBC	NCT03740893
	August, 2019	recruiting	I	leukemia and myelodysplastic syndrome	NCT03770429
	July, 2019	active, not recruiting	II	refractory gastric cancer and metastatic melanoma	NCT03780608
	October, 2019	recruiting	II	resistant prostate cancer	NCT03787680
	October, 2019	recruiting	II	advanced NSCLC	NCT03833440
	December, 2019	recruiting	II	malignant solid tumors	NCT03878095
	November, 2019	recruiting	II	gynaecological cancers	NCT04065269
	July, 2020	recruiting	II	advanced or metastatic *BRCA* mutated breast cancer	NCT04090567
	August, 2020	recruiting	II	ovarian cancer	NCT04239014
	June, 2020	recruiting	II	advanced biliary tract cancer	NCT04298021
	June, 2020	recruiting	II	advanced biliary tract cancer	NCT04298008
	June, 2020	enrolling by invitation	II	relapsed SCLC	NCT04361825
	November, 2020	recruiting	II	recurrent osteosarcoma	NCT04417062
	December, 2020	recruiting	II	advanced solid tumors	NCT04564027
	February, 2021	not yet recruiting	II	extensive stage SCLC	NCT04699838
	February, 2021	not yet recruiting	I	advanced solid tumors expressing HER2 protein or gene	NCT04704661
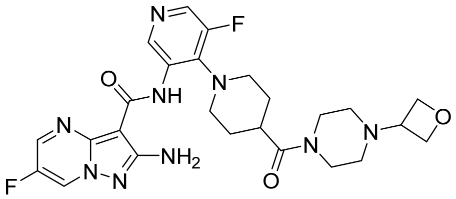 **M4344 (VX-803)**	January, 2015	recruiting	I	advanced solid tumors	NCT02278250
	December, 2020	not yet recruiting	I	PARPi-resistant recurrent ovarian cancer	NCT04149145
	December, 2020	not yet recruiting	I / II	advanced breast cancer	NCT04655183
	July, 2017	recruiting	I	advanced solid tumors, and lymphomas	NCT03188965
	September, 2019	recruiting	I	advanced solid tumors	NCT04095273
	February, 2020	recruiting	I	advanced solid tumors, and ovarian cancer	NCT04267939
	February, 2021	not yet recruiting	I	advanced solid tumors with emphasis on urothelial cancer	NCT04491942
	February, 2021	not yet recruiting	I	advanced solid tumors with emphasis on SCLC, neuroendocrine cancer, and pancreatic cancer	NCT04514497
	March, 2021	not yet recruiting	I	advanced or metastatic cancers of the stomach and intestines	NCT04535401

	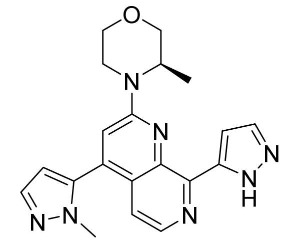	February, 2021	not yet recruiting	I	Recurrent HNSCC	NCT04576091
		February, 2021	not yet recruiting	I	advanced solid tumors, pancreatic cancer, and ovarian cancer	NCT04616534
	**BAY1895344**					
	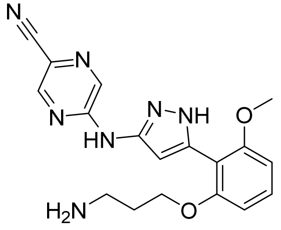	February, 2010	completed	I	advanced and/or metastatic solid tumors	NCT01115790
		June, 2014	completed	I	advanced and/or metastatic solid tumors	NCT02124148
		January, 2015	active, not recruiting	II	*BRCA1/2* mutation associated breast or ovarian cancer, triple negative breast cancer, high grade serous ovarian cancer, and metastatic castrate-resistant prostate cancer	NCT02203513
		October, 2015	completed	I	advanced and/or metastatic solid tumors	NCT02514603
		February, 2016	completed	I	advanced HNSCC	NCT02555644
		May, 2016	active, not recruiting	I	relapsed or refractory acute myeloid leukemia or myelodysplastic syndrome	NCT02649764
		May, 2016	completed	II	extensive stage SCLC	NCT02735980
		September, 2016	completed	I	advanced and/or metastatic solid tumors	NCT02778126
		February, 2017	active, not recruiting	I	recurrent or refractory solid tumors in pediatric patients	NCT02808650
		August, 2016	completed	I	advanced and/or metastatic solid tumors	NCT02860780
		October, 2016	active, not recruiting	II	advanced and/or metastatic solid tumors with RS or HR deficiency	NCT02873975
		March, 2017	active, not recruiting	I	advanced and/or metastatic solid tumors	NCT03057145
**CHK1**	**prexasertib (LY2606368)**	April, 2018	active, not recruiting	II	platinum-resistant or refractory recurrent ovarian cancer	NCT03414047
		May, 2018	active, not recruiting	I	advanced and/or metastatic solid tumors	NCT03495323
		January, 2019	terminated	I	relapsed or refractory acute myeloid leukemia or myelodysplastic syndrome	NCT03735446
		August, 2019	recruiting	I	recurrent refractory or progressive medulloblastoma in pediatric patients	NCT04023669
		September, 2019	recruiting	II	metastatic TNBC	NCT04032080
		September 2019	recruiting	I / II	desmoplastic small round cell tumor and rhabdomyosarcoma	NCT04095221
	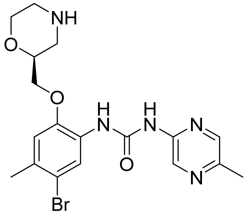	January, 2007	completed	I	solid tumors	NCT00415636
		February, 2009	completed	I / II	pancreatic cancer	NCT00839332
		November, 2009	completed	II	advanced or metastatic NSCLC	NCT00988858
		February, 2011	completed	I / II	NSCLC	NCT01139775
		February, 2011	completed	I	advanced and/or metastatic solid tumors	NCT01296568
		May, 2011	completed	I	advanced and/or metastatic solid tumors	NCT01341457
		May, 2011	completed	I	advanced and/or metastatic solid tumors	NCT01358968
	**rabusertib (LY2603618)**					

	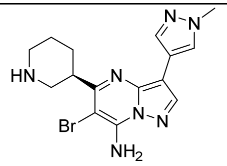 MK-8776 (SCH099776)	October, 2008	completed	I	advanced solid tumors or lymphoma	NCT00779584
		July, 2009	terminated	I	acute leukemias	NCT00907517
		March, 2012	withdrawn	I	advanced solid tumors	NCT01521299
		May, 2013	completed	II	relapsed acute myeloid leukemia	NCT01870596
	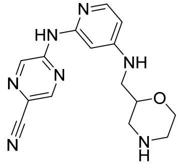 SRA737	July, 2016	completed	I / II	advanced solid tumors	NCT02797977
		July, 2016	completed	I / II	advanced solid tumors or Non-Hodgkin's lymphoma	NCT02797964
	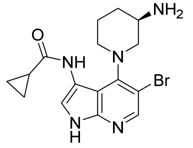 GDC-0575 (ARRY-575)	March, 2012	completed	I	refractory solid tumors or lymphoma	NCT01564251
	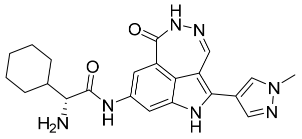 PF-00477736	December, 2006	terminated	I	advanced solid tumors	NCT00437203
	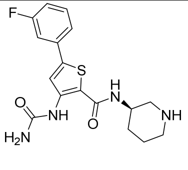 AZD7762	December, 2006	completed	I	advanced solid tumors	NCT00413686
		May, 2007	terminated	I	advanced solid tumors	NCT00473616
		July, 2009	terminated	I	advanced solid tumors	NCT00937664
**WEE1**		February, 2008	completed	I	advanced solid tumors	NCT00648648
		January, 2010	terminated	I	advanced solid tumors	NCT01047007
		May, 2010	terminated	I / II	cervical cancer	NCT01076400
		July, 2010	unknown	II	*TP53* mutated refractory and resistant ovarian cancer	NCT01164995
		July, 2011	completed	II	*TP53* mutated platinum-sensitive ovarian tumors	NCT01357161
		December, 2012	completed	I	refractory solid tumors	NCT01748825
		December, 2013	active, not recruiting	II	advanced solid tumors	NCT01827384
		August, 2013	active, not recruiting	I	newly diagnosed or recurrent glioblastoma	NCT01849146

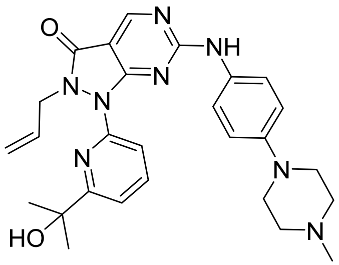

adavosertib (AZD1775/MK-1775)

September, 2013	active, not recruiting	I	newly diagnosed diffuse intrinsic pontine gliomas	NCT01922076
January, 2014	completed	I / II	unresectable adenocarcinoma of the pancreas	NCT02037230
March, 2014	terminated	II	untreated advanced NSCLC	NCT02087241
March, 2014	terminated	II	previously treated NSCLC	NCT02087176
March, 2014	active, not recruiting	I / II	relapsed or refractory solid tumors in pediatric patients	NCT02095132
July, 2014	active, not recruiting	II	recurrent ovarian, primary peritoneal, or fallopian tube cancer	NCT02101775
July, 2014	active, not recruiting	I / II	previously untreated pancreatic cancer that is metastatic or cannot be removed by surgery	NCT02194829
March, 2014	terminated	II	recurrent or metastatic HNSCC	NCT02196168
July, 2014	completed	0	glioblastoma	NCT02207010
January, 2015	active, not recruiting	II	platinum-resistant epithelial ovarian, fallopian tube, or primary peritoneal cancer	NCT02272790
January, 2015	completed	I	advanced solid tumors	NCT02341456
August, 2015	terminated	I	relapsed or refractory myeloid malignancies or untreated acute myeloid leukemia	NCT02381548
January, 2015	recruiting	II	advanced gastric adenocarcinoma harboring *TP53* Mutation	NCT02448329
August, 2015	recruiting	II	advanced refractory solid tumors, lymphomas, or multiple myeloma	NCT02465060
July, 2015	completed	I	advanced solid tumors	NCT02482311
July, 2015	completed	I	HNCSS	NCT02508246
August, 2015	completed	I	refractory solid tumors	NCT02511795
October, 2015	recruiting	II	squamous cell lung cancer	NCT02513563
October, 2016	active, not recruiting	I	muscle invasive bladder cancer	NCT0254661
November, 2015	active, not recruiting	II	metastatic solid tumors	NCT02576444
February, 2016	completed	I	HNSCC	NCT02585973
December, 2015	unknown	II	Relapsed SCLC	NCT02593019
December, 2015	completed	I	advanced or metastatic solid tumors	NCT02610075
December, 2015	active, not recruiting	I	advanced solid tumors	NCT02617277
February, 2016	recruiting	early I	advanced high grade ovarian, fallopian tube, or primary peritoneal cancer	NCT02659241
May, 2017	completed	II	advanced acute myeloid leukemia or myelodysplastic syndrome	NCT02666950
June, 2016	terminated	II	relapsed SCLC with MYC amplification, *CDKN2A* mutation and *TP53* mutation	NCT02688907
May, 2017	withdrawn	I	relapsed or refractory acute myeloid leukemia in pediatric patients	NCT02791919
August, 2016	recruiting	I / II	heamotologic or solid malignancies in pediatric patients	NCT02813135
September, 2016	completed	I	metastatic colorectal cancer	NCT02906059
November, 2016	active, not recruiting	II	platinum-refractory extensive-stage SCLC	NCT02937818
January, 2017	active, not recruiting	II	metastatic TNBC	NCT03012477
June, 2017	active, not recruiting	I	HNSCC	NCT03028766
September, 2018	suspended	II	advanced refractory solid tumors with CCNE1 amplification	NCT03253679
May, 2018	recruiting	II	advanced solid tumors with loss of SETD2	NCT03284385
October, 2017	completed	I	advanced solid tumors	NCT03313557

September, 2017	completed	I	advanced solid tumors	NCT03315091
February, 2018	active, not recruiting	II	metastatic TNBC	NCT03330847
December, 2017	completed	I	advanced solid tumors	NCT03333824
October, 2017	active, not recruiting	I	cervical, upper vaginal and uterine cancers	NCT03345784
December, 2018	recruiting	II	recurrent ovarian, primary peritoneal, or fallopian tube cancer	NCT03579316
October, 2018	recruiting	II	recurrent or persistent uterine cancer	NCT03668340
May, 2019	terminated	II	advanced AML, myelodysplastic syndrome and myelofibrosis	NCT03718143
April, 2020	recruiting	I	advanced solid tumors with PARPi resistance	NCT04197713
March, 2017	active, not recruiting	II	dvanced refractory solid tumors, lymphomas, and multiple myelomas, with *BRCA* mutations	NCT04439227
June, 2020	recruiting	I	advanced solid tumors	NCT04462952
October, 2020	recruiting	I	incurable esophageal and gastroesophageal junction cancers	NCT04460937
November, 2020	recruiting	II	uterine serous carcinoma	NCT04590248

Aligned according to NCT numbers from the lowest to highest. AML – acute myeloid leukemia; HNSCC – head and neck squamous cell carcinoma; HR – homologous recombination; NSCL – non-small cell lung carcinoma; RS – replication stress; SCLC – small cell lung carcinoma; TNBC – triple negative breast cancer
